# Thalidomide Prevents Alcoholic Liver Injury in Rats Through Inhibition of Kupffer Cell Sensitization

**DOI:** 10.1186/1476-5926-2-S1-S37

**Published:** 2004-01-14

**Authors:** Nobuyuki Enomoto, Yoshiyuki Takei, Miyoko Hirose, Kenichi Ikejima, Tsuneo Kitamura, Nobuhiro Sato

**Affiliations:** 1 Department of Gastroenterology, Juntendo University School of Medicine, 2-1-1 Hongo, Bunkyo-ku, Tokyo 113-8421, Japan

## Introduction

Increasing lines of evidence show that activation of Kupffer cells plays a pivotal role in initiation and progression of alcoholic liver disease. Kupffer cell activation is evoked by endotoxin (lipopolysaccharide, LPS), which leads to rapid elevation of intracellular calcium accompanied by release of a variety of mediators including cytokines, eicosanoids and reactive radical species. Among them, TNF-alpha is a critical factor in the pathogenesis of alcoholic liver disease.

Thalidomide (alpha-N-phthalimidoglutarimide) was initially used as a sedative and antiemetic during pregnancy but was withdrawn from the market due to its teratogenic effects. Before it was banned, thalidomide was recognized to reduce dramatically symptoms associated with erythema nodosum leprosum, a complication of Hansen's disease. Subsequently, it was shown that thalidomide suppresses TNF-alpha production by macrophages and other cell types such as activated T cells [1,2], which was attributed to a mechanism of thalidomide action [3].

Accordingly, the purpose of this study was to determine whether thalidomide could prevent alcohol-induced liver injury. Here we report that thalidomide prevents liver damage caused by chronic ethanol exposure through not only suppression of TNF-alpha production but abrogation of Kupffer cell sensitization to LPS.

## Methods

### Animals and Treatments

In this study, a model of alcoholic liver injury based on the sensitization of Kupffer cells, in which rats are given ethanol (5 g/kg body wt) once every 24 hours [4], was used. This model achieves inflammatory and necrotic changes in the liver only in 8 weeks, which mimics features of clinical alcohol liver injury [5]. Liver damage was evaluated after 8 weeks of ethanol since histological manifestations are preceded by sensitization of Kupffer cells to LPS treatment, and Kupffer cell sensitization to LPS was evaluated at 4 weeks. Female Wistar rats weighing 200–250 g were fed on a liquid diet (Oriental, Tokyo, Japan). Two groups of rats received an oral dose of thalidomide (5 mg/kg body wt) only or concurrently with ethanol [6]. Gut permeability was measured in isolated segments of ileum from translocation of horseradish peroxidase as described previously [6].

### Kupffer Cell Preparation Culture, Measurement of [Ca^2+^]_i_, and TNF-alpha Production

Kupffer cells were isolated by collagenase digestion and [Ca^2+^]_i _was measured using a microspectrofluorometer with fura-2, and TNF-alpha in the culture media was measured using an enzyme-linked immunosorbent assay (ELISA) kit (Genzyme, Cambridge, MA).

### Statistical Analysis

All results were expressed as mean – S.E.M. Statistical differences between means were determined using analysis of variance (ANOVA) or ANOVA on ranks or Bonferroni's post-hoc test or Student's *t *test as appropriate. p &lt; 0.05 was selected prior to the study to reflect significance.

## Results

### Effect of Thalidomide on Alcoholic Liver Injury

Ethanol administration once every 24 h for 8 weeks caused pronounced pathological changes in the liver lobule, including steatosis, necrosis and inflammation in the liver, confirming our earlier results. In marked contrast, these pathological changes were prevented almost completely by coadministration of thalidomide (5 mg/kg). Likewise, ALT was increased to 91 – 7 IU/L in the 8-week ethanol group, which was three times higher than the values in the control, non-treated rats (Table 1). The increase in ALT was prevented completely by thalidomide. Similar results were obtained for AST values.

**Table 1:** Effect of thalidomide on ethanol-induced liver injury

	ALT(IU/L)	AST(IU/L)
Control	101 – 12	30 – 6
Thalido (5 mg/kg) 8 weeks	62 – 3	38 – 6
Ethanol (5 g/kg) 8 weeks	162 – 7*	91 – 7*
Ethanol + Thalidomide 8 weeks	126 – 15**	37 – 3**

* p &lt; 0.05 vs. control, ** p &lt; 0.05 vs. 8 weeks of ethanol

### Effect of Thalidomide on Ethanol plus LPS-Induced Liver Injury

In an attempt to evaluate the sensitization of Kupffer cells to LPS *in vivo*, LPS (5 mg/kg) was administered i.v. and liver histology were evaluated 24 hours later (data not shown). LPS caused focal necrosis with neutrophil infiltration in the control liver. In the group treated with thalidomide for 4 weeks, liver histology revealed only slight infiltration of neutrophils, but lacking evidence of necrosis. This result agrees with serum transaminase levels, which were slightly lower in the thalidomide-only group. In the 4-week ethanol group, LPS injection resulted in marked aggravation of these pathological parameters. Moreover, steatosis was prominent in this group. Treatment with thalidomide dramatically improved liver injury, which exhibited near normal histology. The increased ALT levels observed 24 hours after LPS injection in the 4-week ethanol group was blocked completely by thalidomide. Similar results were obtained with AST values (Table 2).

**Table 2:** Effect of thalidomide on ethanol plus LPS-induced liver injury.

	AST(IU/L)	ALT(IU/L)
LPS(5 mg/ml)	846 – 227	483 – 167
Thalido 4 weeks + LPS	307 – 15	239 – 15
Ethanol 4 weeks + LPS	2091 – 295*	1200 – 375*
Ethanol + Thalido 4 weeks + LPS	379 – 231**	219 – 179**

* p &lt; 0.05 vs. LPS, ** p &lt; 0.05 vs. 4 weeks of ethanol + LPS.

### Effect of Thalidomide and Ethanol on LPS-Induced Increases in [Ca^2+^]_i _and TNF-alpha Production in Isolated Kupffer Cells

To further determine the effect of thalidomide of ethanol-induced Kupffer cell sensitization to LPS, LPS-induced increase of [Ca^2+^]_i _and LPS-induced production of TNF-alpha were measured. As reported earlier, LPS evoked a transient increase in [Ca^2+^]_i _of Kupffer cells obtained from control rats from basal levels to 81 – 13 nM. After the peak increase, [Ca^2+^]_i _declined rapidly returning to basal value. Thalidomide treatment did not alter the [Ca^2+^]_i _response. In contrast, the LPS-induced peak [Ca^2+^]_i _elevation was about 2 to 3-fold greater in Kupffer cells isolated from rats given ethanol for 4 weeks. It was also noted that, after the peak increase, [Ca^2+^]_i _started to decrease but remained elevated over 180 sec. The increased [Ca^2+^]_i _response was blocked completely by concurrent administration of thalidomide with ethanol for 4 weeks. Moreover, TNF-alpha production shows similar pattern (Table 3).

**Table 3:** Effect of ethanol and thalidomide on LPS-induced increases in intracellular Ca^2+ ^and TNF-alpha production in isolated Kupffer cells.

	[Ca^2+^]_i _(nM)	TNF-alpha(pg/ml)
LPS (100 ng/ml)	85 – 4	559 – 71
Thalido 4 weeks + LPS	92 – 6	578 – 77
Ethanol 4 weeks + LPS	227 – 26*	1104 – 110*
Ethanol + Thalido 4 weeks + LPS	83 – 4**	625 – 72**

* p &lt; 0.05 vs. control, ** p &lt; 0.05 vs. 4 weeks of ethanol.

### Effect of Ethanol and Thalidomide on CD14 Expression in the Liver

Because CD14, a functional LPS/LBP receptor, is critical for signaling pathways leading to expression of cytokines, eicosanoids and radical species in Kupffer cells, we measured CD14 with Western blotting (data not shown). Liver from control rats expressed the 55 kD CD14. Thalidomide did not change the amount of CD14 protein; however, the band was far more intense in liver from rats treated with ethanol chronically. Furthermore, the ethanol-induced increase in CD14 protein levels was abrogated completely with co-administration of thalidomide for 4 weeks.

### Effects of Ethanol and Thalidomide on Gut Permeability

As reported previously, Kupffer cell sensitization to LPS is caused by LPS. Therefore, we determined the effect of thalidomide on gut-permeability (Table 4). Thalidomide alone did not alter the basal value of gut permeability. In marked contrast, two hours after the final ethanol treatment in the 4 weeks group, gut permeability was increased 10-fold as compared to control values. The ethanol-induced increase in gut permeability was, however, not affected by treatment with thalidomide, As expected, LPS levels in portal blood were not different between the ethanol-only and ethanol + thalidomide groups (140 – 51 pg/ml vs. 99 – 40 pg/ml, N.S.).

**Table 4:** Effect of ethanol and thalidomide treatment on gut permeability.

	HRP(U/L)
Control	63 – 6
Thalido 4 weeks	68 – 12
Ethanol 4 weeks	591 – 90*
Ethanol + Thalido 4 weeks	543 – 161*

* p &lt; 0.05 vs. control.

### Effect of Thalidomide on LPS-Induced TNF-alpha mRNA Expression and TNF-alpha Production in Cultured Kupffer Cells

After addition of LPS (100 ng/ml) to Kupffer cells isolated from the untreated, control rats, the amount of TNF-alpha mRNA increased and reached a maximal value after 1 hour, followed by a gradual decline over 2 hours (data not shown). Inclusion of thalidomide (5 micromolar) in the culture media did not alter the peak level of TNF-alpha mRNA occurring at 1 hour after LPS. However, 3 hours after LPS addition, the amount of TNF-alpha mRNA in Kupffer cells treated with thalidomide was 30% lower than that of control Kupffer cells, indicating that thalidomide accelerated decrease in TNF-alpha mRNA (Table 5A). Thalidomide (5 micromolar) reduced production of TNF-alpha by Kupffer cells challenged with LPS (100 ng/ml) for 4 hours (Table 5B).

**Table 5:** Effect of thalidomide on LPS-induced TNF-alpha mRNA expression and TNF-alpha production in cultured Kupffer cells.

**(A) **TNF-alpha and beta-actin mRNA expression	
	TNF-alpha/beta-actin(%1 h LPS)

LPS (100 ng/ml) 1 hour	100
LPS + Thalido (5 micromolar) 1 hour	97
LPS 3 hours	65
LPS + Thalido 3 hours	41

**(B) **TNF-alpha production	

	TNF-alpha (pg/ml)

LPS (100 ng/ml)	501 – 19
LPS + Thalido (5 micromolar)	268 – 4*

* p &lt; 0.05 vs. control.

### Effect of Thalidomide and TNF-alpha on Expression of CD14 in Cultured Kupffer Cells

To explore the mechanism by which thalidomide abrogated Kupffer cell sensitization to LPS caused by chronic ethanol, we determined the effect of TNF-alpha on CD14 expression in Kupffer cells obtained from normal rats *in vitro *(data not shown). Treatment with TNF-alpha (10 ng/ml) for 24 hours resulted in enhancement of CD14 expression in Kupffer cells. Inclusion of thalidomide (5 micromolar) in the culture media reduced CD14 expression to a level comparable to the control.

### Effect of Thalidomide and TNF-alpha on LPS-Induced TNF-alpha Production by Cultured Kupffer Cells

LPS-induced TNF-alpha production by isolated Kupffer cells was compared between groups cultured for 24 hours in the presence or absence of TNF-alpha (10 ng/ml) prior to LPS challenge. Kupffer cells pretreated with TNF-alpha produced about 30% more TNF-alpha in response to LPS (100 ng/ml) than the control cells cultured in the absence of TNF-alpha (Table 6). Thalidomide (5 micromolar) completely blocked the TNF-alpha-induced enhancement of TNF-alpha production by Kupffer cells in response to LPS.

**Table 6:** Effect of thalidomide and TNF-alpha on LPS-induced TNF-alpha production by cultured Kupffer cells.

	TNF-alpha (pg/ml)
LPS (100 ng/ml)	726 – 82
TNF-alpha (10 ng/ml)+ LPS	936 – 186*
Thalidomide (5 micromolar) + TNF-alpha + LPS	439 – 105**

* p &lt; 0.05 vs. TNF-alpha(-), ** p &lt; 0.05 vs. TNF-alpha(+).

## Discussion

The results of this study showed that thalidomide prevented liver injury caused by chronic ethanol treatment (Table 1). To investigate the mechanism of inhibitory effects of thalidomide against ethanol-induced liver injury, we determined if thalidomide directly acted on Kupffer cells thereby reducing TNF-alpha production. As shown in Table 6, thalidomide did not affect the levels of TNF-alpha mRNA in Kupffer cells after 1 hour of challenged with LPS, however, reduced later the amounts of TNF-alpha mRNA. Consequently, the LPS-induced TNF-alpha synthesis by Kupffer cells was inhibited by thalidomide. This result agrees with an earlier work showing that thalidomide abrogated LPS-induced TNF-alpha production from macrophages [1,2]. It was reported that thalidomide decreases TNF-alpha production by accelerating the degradation of its mRNA [2].

TNF-alpha has a pleiotropic cytokine and regulatory mechanisms of TNF-alpha production in macrophages and its intracellular signaling have been studied extensively [7]. It was suggested that TNF-alpha acts on macrophages/monocytes to promote its own synthesis and secretion [8]. The fact that thalidomide destabilizes TNF-alpha mRNA provides a good basis for the use of thalidomide to treat alcohol-induced liver injury because a recent work by Kishore et al. revealed stabilization of TNF-alpha mRNA by chronic ethanol [9]. We hypothesized that an autocrine stimulation by TNF-alpha is involved in the ethanol-induced Kupffer cell sensitization and that thalidomide interferes with this pathway, leading to abrogation of Kupffer cell sensitization.

This scenario is supported by the facts that pretreatment with TNF-alpha led to an increased TNF-alpha synthesis in response to LPS and enhancement of CD14 expression in Kupffer cells, and that inclusion of thalidomide in the culture media inhibited this TNF-alpha-induced autocrine activation of TNF-alpha synthesis (Table 6). Furthermore, thalidomide blocked completely the TNF-alpha-induced upregulation of CD14 in Kupffer cells *in vitro*.

The precise mechanisms of the thalidomide actions remain to be pursued, however, the results of this study suggest that thalidomide prevents liver injury caused by chronic ethanol consumption through destabilization of TNF-alpha mRNA and abolishing Kupffer cell sensitization to LPS. Though currently its side effects preclude the use of thalidomide in clinical settings and require rigors of the drug distribution program and prescription decision, efforts to synthesize thalidomide analogues lacking teratogenic effects but having more potent efficacy are underway [10]. Given that thalidomide has unique mechanisms of actions, there appears to be a strong possibility that this type of drug will prove beneficial to patients with severe alcoholic liver injury.
